# The complete chloroplast genome of the fern *Asplenium tenerum* (Aspleniaceae)

**DOI:** 10.1080/23802359.2020.1831985

**Published:** 2020-11-11

**Authors:** Yang Peng, Suzhou Zhang, Shanshan Dong, Tao Peng

**Affiliations:** a Laboratory of Southern Subtropical Plant Diversity, Fairy Lake Botanical Garden, Shenzhen and Chinese Academy of Sciences, Shenzhen, China; b School of Life Sciences, Guizhou Normal University, Guiyang, China

**Keywords:** Plastome, *Asplenium tenerum*, Polypodiales

## Abstract

Plastid genomes are useful markers in resolving plant phylogenetic relationships for various taxonomic groups. Here, we sequenced and *de novo* assembled the complete plastid genome sequence of the fern *Asplenium tenerum* Forst. (Aspleniaceae, Polypodiales) using the genome skimming data. The newly generated plastid genome is conserved in structure and gene content compared with that of closely related species. Plastid phylogenetic analysis of Polypodiales ferns recovered a robust phylogeny, supporting the close relationship of *A. tenerum* with *Asplenium prolongatum*.

*Asplenium* L. (Aspleniaceae, Polypodiales), with about 720 species (Kramer and Viane 1990), is a cosmopolitan genus with its center of diversity mostly in tropics. This genus is characterized by linear or oblong sori borne obliquely on the upper side of a veinlet. The morphology of the species within this genus varied considerably. Frequent hybridization (Werth et al. 1985) and resultant morphological homoplasy and overlap further confused the species delimitation (Shepherd et al. 2008). Plastid sequences are widely used in molecular phylogenetic studies of ferns (Lu et al. 2015; Wei et al. 2017; Kuo et al. 2018) due to their easy accessibility, relatively fast substitution rate, and clear orthology. Contributing plastid genome sequences would be important for further illuminating and improving the global fern phylogeny, especially for the phylogenetic relationships of the highly diversified Polypodiales species.

Fresh leaves of *Asplenium tenerum* Forst. were collected from the Plant Conservation Center of Fairy Lake Botanical Garden, Shenzhen, Guangdong, China (22°35′49″N, 114°10′5″E). The voucher specimen has been deposited at SZG (Herbarium of Shenzhen Fairy lake Botanical Garden, Shenzhen, China), with collection number DongSS20150706. DNA extraction was performed with the NucleoSpin Plant II Midi DNA extraction kit (Macherey-Nagel, Düren, Germany). The DNA quality and quantity of each sample were examined using 1% Agarose gel electrophoresis, Qubit fluorometer (Invitrogen), and NanoDrop 2000 spectrophotometer. For genomic DNA sequencing, 1 µg high quality genomic DNA was sheared using the Covaris M220 (Woburn, MA, USA), DNA fragments of 300–500 bp were used to generate sequencing libraries using the Illumina TruSeqTM DNA PCR-free library preparation kit (Illumina, CA, USA) following the manufacturer’s instructions. The libraries were paired-end (2 × 150 bp) sequenced on an Illumina HiSeq 2000 sequencing platform at the WuXi NextCode (Shanghai, China). Approximately 6 Gb sequencing data were generated for the sample, the sequence reads have been submitted to GenBank SRA database under the Accession No. SRR9897626. The raw NGS data were trimmed and filtered for adaptors, low quality reads, undersized inserts, and duplicate reads using Trimmomatic (Bolger et al. 2014). The resultant clean reads were *de novo* assembled using CLC Genomics Workbench v5.5 (CLC Bio, Aarhus, Denmark). The assembled contigs were blasted to the plastid genome sequences of other *Asplenium* species that downloaded from GenBank, yielding a complete circular plastid genome sequence of 154,628 bp. The plastome of *A. tenerum* was annotated in Geneious v10.0.2 (www.geneious.com) by transferring annotations after aligning the newly generated sequence with those of the other plastomes from the same genus.

The complete plastid genome of *A. tenerum* (GenBank accession no. MT700551) is 154,628 bp in length with an overall GC content of 40.82%. The genome displays a typical quadripartite structure consisting a small single-copy region (SSC; 21,374 b p), a large single-copy region (LSC; 81,205 bp), and a pair of identical inverted repeats (IR; 26,117 bp). The genome encoded a non-redundant gene set similar to that of the other Aspleniaceae plastomes, including 84 protein-coding genes, eight rRNA genes, and 34 tRNA genes. Nine protein-coding genes (*ndhA*, *rpl2*, *rpl16*, *petD*, *petB*, *rpoC1*, *atpF*, *rps16*, and *ndhB*) were disrupted by one intron, and three genes (*clpP*, *rps12*, and *ycf3*) by two, including the trans-spliced *rps12* gene.

Plastid phylogenetic analysis was performed using all 81 available plastomes of Polypodiales (as of June 2020) together with our newly generated plastid sequence of *A. tenerum*. Each of the conserved 75 protein-coding genes was extracted from a total of 82 Polypodiales species, aligned, and concatenated in Geneious v10.0.2 separately. The concatenated protein-coding gene dataset (Figshare database under DOI: https://doi.org/10.6084/m9.figshare.12853019.v2) was analyzed using RAxML v7.2.3 (Stamatakis 2006) for phylogenetic reconstructions with the maximum-likelihood (ML) method with 1000 fast bootstrap replicates under the GTRGAMMA nucleotide substitution model. The resultant phylogenetic tree (Figure 1) is consistent with previous plastid phylogenomic studies of ferns (Kuo et al. 2018), and suggested a close relationship of *A. tenerum* and *Asplenium pekinense*.

**Figure 1.**
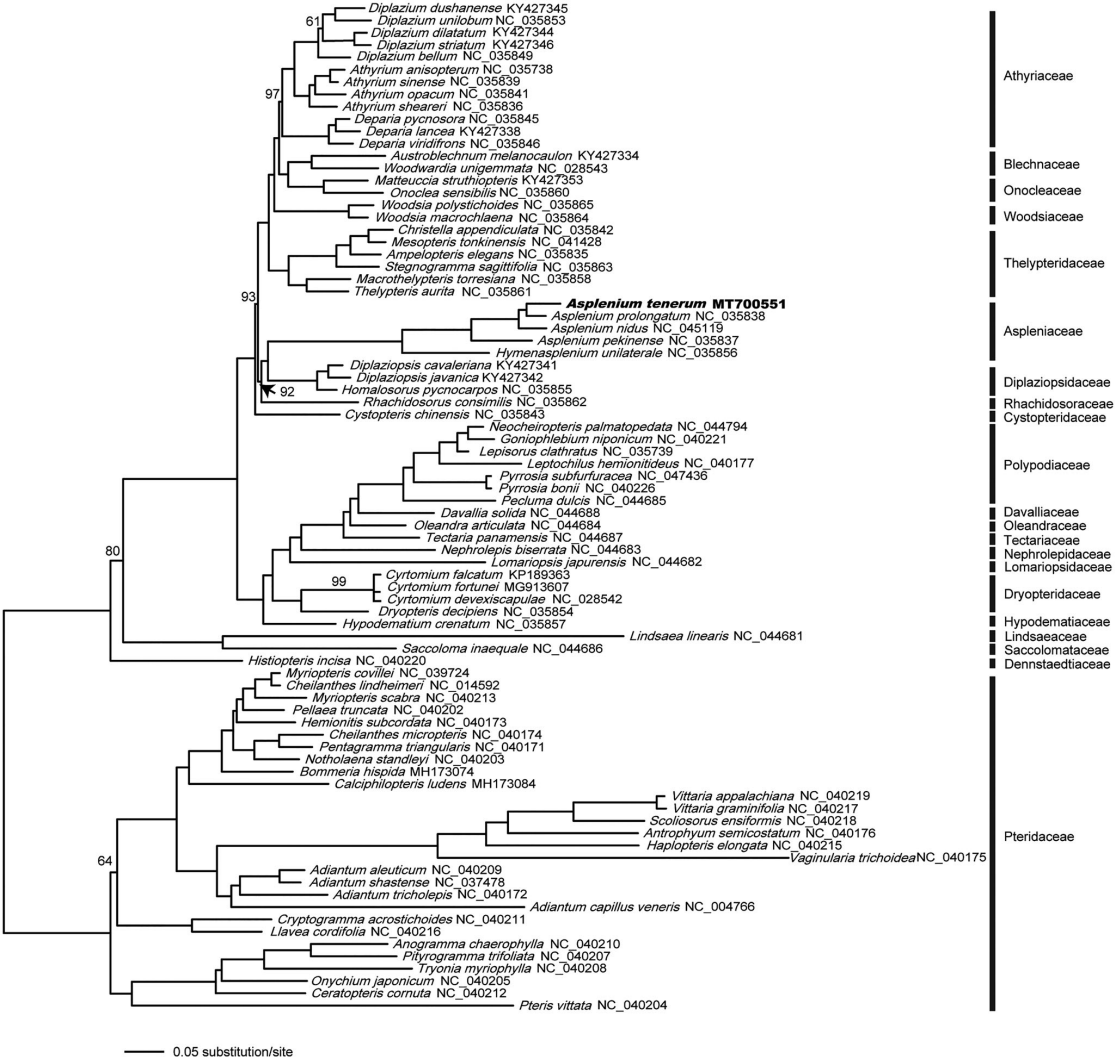
The phylogenetic relationship of *A. tenerum* and 81 Polypodiales species, with the family Pteridaceae rooted as the outgroup. The numbers above the branches are bootstrap support values, all branches maximally supported unless otherwise marked. The newly sequenced *A. tenerum* is in bold.

## Data Availability

The complete plastid genome of *Asplenium tenerum* newly generated in current study have been submitted to GenBank under the accession number of MT700551. The *Asplenium tenerum* genome sequencing reads have been deposited in the Short Read Achieve (SRA) database of NCBI under the accession number of SRR9897626. The data matrix used for current phylogenetic reconstruction can be accessed from the Figshare database under DOI: https://doi.org/10.6084/m9.figshare.12853019.v2.
